# Risk factors for hepatic hydrothorax in patients with cirrhosis: a clinical retrospective study

**DOI:** 10.3389/fmed.2023.1165604

**Published:** 2023-06-01

**Authors:** Xue Bai, Xiaoyan Liu, Yanhui Shi, Wenwen Li, Qiang Li, Wenjun Du

**Affiliations:** ^1^Cheeloo College of Medicine, Shandong University, Jinan, China; ^2^Department of Liver Diseases, Shandong Public Health Clinical Center, Shandong University, Jinan, China; ^3^ICON Plc, Beijing, China

**Keywords:** liver cirrhosis, hepatic hydrothorax, risk factors, MELD scores, portal vein

## Abstract

**Aims and background:**

Hepatic hydrothorax, which presents as an unexplained pleural effusion, is one of the important complications in patients with end-stage cirrhosis. It has a significant correlation with prognosis and mortality. The aim of this clinical study was to detect the risk factors for hepatic hydrothorax in patients with cirrhosis and to better understand potentially life-threatening complications.

**Methods:**

Retrospectively, 978 cirrhotic patients who were hospitalized at the Shandong Public Health Clinical Center from 2013 to 2021 were involved in this study. They were divided into the observation group and the control group based on the presence of hepatic hydrothorax. The epidemiological, clinical, laboratory, and radiological characteristics of the patients were collected and analyzed. ROC curves were used to evaluate the forecasting ability of the candidate model. Furthermore, 487 cases in the experimental group were divided into left, right, and bilateral groups, and the data were analyzed.

**Results:**

The patients in the observation group had a higher proportion of upper gastrointestinal bleeding (UGIB), a history of spleen surgery, and a higher model for end-stage liver disease (MELD) scores compared with the control group. The width of the portal vein (PVW) (*P* = 0.022), prothrombin activity (PTA) (*P* = 0.012), D-dimer (*P* = 0.010), immunoglobulin G (IgG) (*P* = 0.007), high-density lipoprotein cholesterol (HDL) (*P* = 0.022), and the MELD score were significantly associated with the occurrence of the hepatic hydrothorax. The AUC of the candidate model was 0.805 (*P* < 0.001, 95% CI = 0.758–0.851). Portal vein thrombosis was more common in bilateral pleural effusion compared with the left and right sides (*P* = 0.018).

**Conclusion:**

The occurrence of hepatic hydrothorax has a close relationship with lower HDL, PTA, and higher PVW, D-dimer, IgG, and MELD scores. Portal vein thrombosis is more common in cirrhotic patients with bilateral pleural effusion compared to those with unilateral pleural effusion.

## Introduction

Hepatic hydrothorax (HH) is an important complication of decompensated cirrhosis and portal hypertension. It is a pleural effusion, typically of more than 500 mL, in patients with liver cirrhosis that do not present with coexisting underlying cardiac or pulmonary diseases (1, 2). The occurrence of HH has been reported in 4–16% of patients with cirrhosis. Most patients with HH have ascites in combination, of which 59–85% are right-sided, 12–17% are left-sided, and 2–29% are bilateral (1, 3–7). There were very few reported cases of HH without ascites (8, 9). HH was first reported by Morrow et al. (10) while describing a rapid accumulation of massive right pleural effusion; however, the pathogenesis is still unclear. One of the well-accepted theories is that a “positive” intra-abdominal pressure and a “negative” intrathoracic pressure of the pleural cavity cause ascites to enter the thoracic cavity through a physiological defect in the diaphragm (11–14). HH has a close relationship with poor prognosis (15). Once HH appears, liver transplantation is considered to be the most specific and effective treatment for those with refractory disease. In addition to diuretics, sodium restriction, and therapeutic thoracentesis (15–18), the use of trans-jugular intrahepatic portosystemic shunts (TIPSs) and surgical repair of diaphragmatic defects relieve the symptoms of HH but are usually limited in clinical practice due to economic factors (19, 20).

Previous studies have suggested some factors that are related to the occurrence of HH. Hou et al. claimed HH was positively associated with moderate-large ascites, Child–Pugh class B–C, lower albumin (Alb), higher prothrombin time (PT), and international normalized ratio (INR) (21). Deleuran et al. and Matei et al. indicated that bilirubin, diabetes, and non-use of non-selective beta-blockers were risk factors for hepatic hydrothorax (7, 22). Based on the abovementioned studies, we retrospectively analyzed the epidemiological characteristics, clinical manifestations, and laboratory indicators of cirrhotic patients with pleural effusion and tried to explore more risk factors of HH, in order to better understand these complications of cirrhosis.

## Materials and methods

### Patients and setting

**Figure 1:**
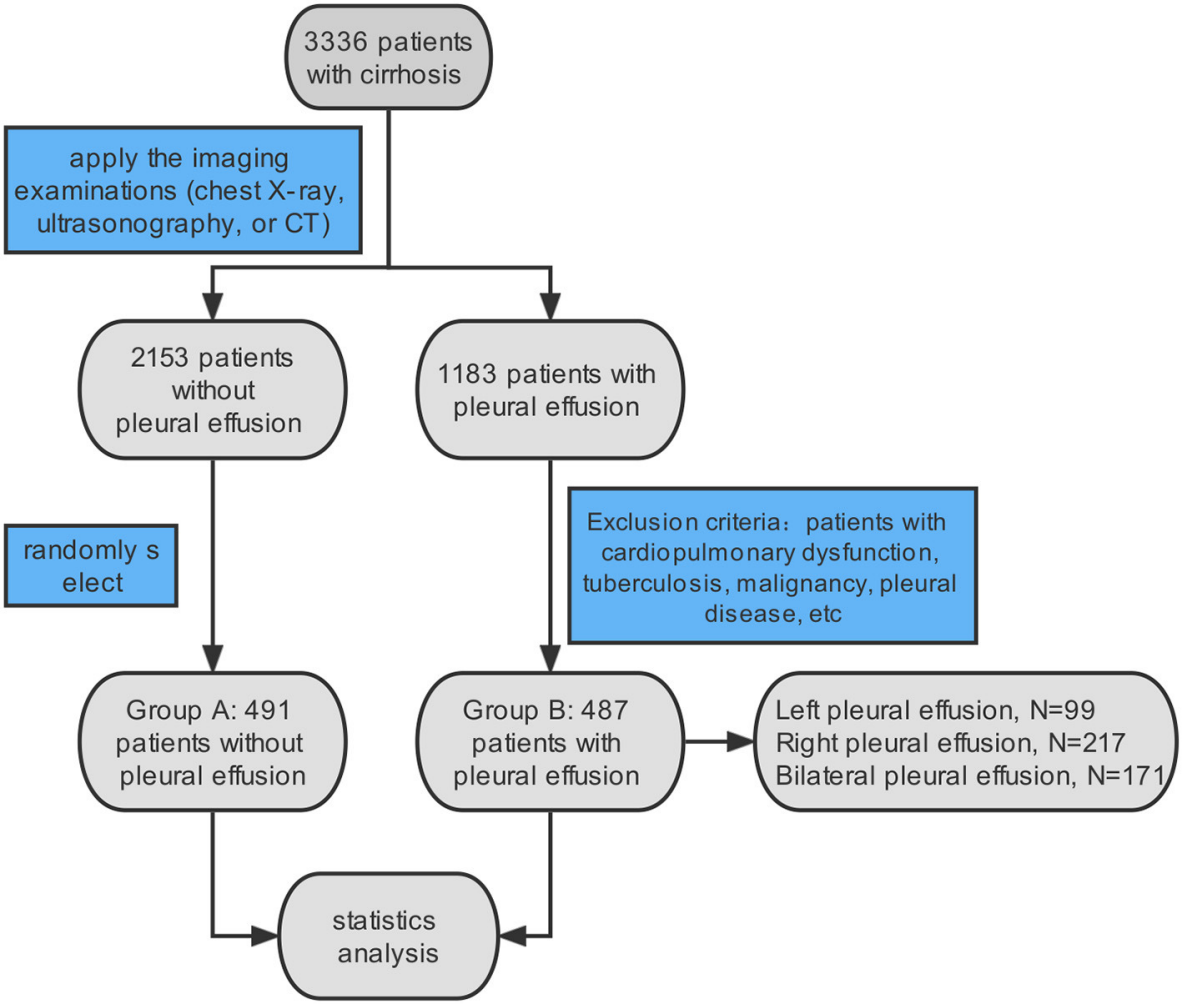
Flow chart of selecting patients. A total of 3,336 patients with cirrhosis and ascites were identified according to the ICD 10 diagnosis. In total of 487 patients were included in our study, meeting the imaging examination and exclusion criteria.

By using electronic medical records, a total of 3,336 patients with cirrhosis, including 1,183 cases with pleural effusion and 2,153 cases without pleural effusion, who were hospitalized at the Shandong Public Health Clinical Center from 2013 to 2021 were involved in this study. All patients matched the International Classification of Diseases, 10th revision (ICD-10) codes for liver cirrhosis (ICD-10: K74.1–K74.6/K70.300) and ascites (R18). For patients with repeated hospitalizations during 2013–2021, only their first hospitalization, which satisfied the above criteria, was entered. Of these, 1,183 patients had confirmed pleural effusion by relevant imaging examinations (chest X-ray, ultrasonography, or CT). Finally, a total of 487 patients with HH were enrolled in our study as the observation group. Similarly, 491 patients were randomly selected from the remaining 2,153 samples without pleural effusion as the control group by applying the random number generator in SPSS (Figure 1). The patients in the two met all the criteria and the exclusion criteria were as follows: (1) cardiopulmonary dysfunction (ICD-10: I00-I99, J00-J89, J95-J97), (2) tuberculosis (ICD-10: A15-A16), (3) malignancies (ICD-10: C00-C97), (4) pleural disease (ICD-10: J90-J94), and (5) patients with TIPS (ICD-9-CM: 39.1). Among the 487 cases with HH, there were 99 cases with left pleural effusions, 217 cases with right pleural effusions, and 171 cases with bilateral pleural effusions.

### Data collection

We analyzed the epidemiological characteristics, clinical manifestations, and laboratory findings of patients from each group, including demographic characteristics, etiology, complications or combination diseases, operation history, blood transfusion history, and laboratory tests. Demographic characteristics comprised age and sex. Etiology involved hepatitis B cirrhosis, hepatitis C cirrhosis, alcoholic cirrhosis, autoimmune hepatitis (AIH), primary biliary cirrhosis (PBC), and cirrhosis caused by unknown factors. Complications or combination diseases involved upper gastrointestinal bleeding, esophageal and gastric fundal varices, hepatic encephalopathy, bacterial peritonitis, portal vein thrombosis, hypertension, and diabetes. Operation history included splenectomy or splenic embolization. Laboratory findings included alanine aminotransferase (ALT), aspartate aminotransferase (AST), γ-glutamyl transpeptidase (GGT), albumin (Alb), total cholesterol (TC), triglyceride (TG), high-density lipoprotein cholesterol (HDL-c), creatinine (Cr), white blood cell count (WBC), hemoglobin (Hb), platelet count (PLT), prothrombin activity (PTA), D-dimer, immunoglobulin G (IgG), interleukin-6 (IL-6), C reactive protein (CRP), and serum amyloid A (SAA). The width of the portal vein (PVW) by ultrasonic examination was also taken into account in the study. The examinations for diagnosis of portal vein thrombosis were enhanced by CT or magnetic resonance imaging. Liver disease severity was estimated using the Child–Pugh stage and the model of end-stage liver disease (MELD) score. The abovementioned data were the first lab test results of the patients after the diagnosis of HH during this hospitalization.

### Statistics analysis

Clinical parameters were evaluated using the chi-square test for discrete variables and the *t*-test or Mann–Whitney *U-*test for continuous variables. Multiple logistic regression analyses were performed to identify the factors associated with the occurrence of HH. For the regression results, 30% of the total sample was randomly selected as the validation group to verify the statistical results. The Kruskal–Wallis test was used for comparison among the three groups of left, right, and bilateral pleural effusions. A *P*-value of <0.05 was considered statistically significant. All data analyses were performed using SPSS v. 25.0 (SPSS Inc., Chicago, IL, USA).

## Results

### Baseline characteristics in the HH group compared with the control group

A total of 988 cirrhotic inpatients were enrolled in this study, including 487 cases with HH as the observation group and 491 cases without HH as the control group. There were 358 male subjects in the observation group and 339 male subjects in the control group (*P* = 0.107). There was no difference between the two groups in the proportion of those aged between 40 and 60 years (58.3 vs. 55.1%, *P* = 0.472). The predominant etiology in both groups was HBV infection (73.5 vs. 75.0%, *P* = 0.756). Complications or combination diseases, including hypertension, diabetes, esophagogastric varices (EGV), hepatic encephalopathy (HE), and portal vein thrombosis showed no difference in the two groups. However, more individuals in the HH group had a history of spleen surgery (splenectomy or splenic embolization) compared with the control group (10.9 vs. 4.9%, *P* = 0.000). MELD scores and Child–Pugh scores were also assessed in both groups, and the results showed that the proportion of high MELD scores was higher in the observation group compared with the control group (*P* = 0.038) (Table 1).

**Table 1:** Baseline characteristics in the HH group compared with the control group.

**Variables**	**Without HH**	**HH**	**Z/χ2**	***P***
	**N** = **491**	**N** = **487**		
Sex (male/female) n	339/153	358/129	2.599	0.107
Age *n* (%)			0.015	0.472
<40	41 (8.3%)	43 (8.8%)		
40–60	271 (55.1%)	284 (58.3%)		
>60	180 (36.6%)	160 (32.9%)		
Etiology *n* (%)			4.205	0.756
HBV	357 (75.0%)	349 (73.5%)		
HCV	10 (2.1%)	8 (1.7%)		
PBC	18 (3.8%)	20 (4.2%)		
AIH	7 (1.5%)	6 (1.3%)		
Alcoholic liver	49 (10.3%)	51 (10.7%)		
Unknown reason	50 (10.4%)	53 (10.9%)		
**Complication/combination disease** ***n*** **(%)**			
Hypertension	89 (18.1%)	108 (22.2%)	2.544	0.111
Diabetes	84 (17.1%)	79 (16.2%)	0.128	0.721
UGIB	35 (7.1%)	54 (11.1%)	4.678	**0.031**
EGV	144 (29.3%)	165 (33.9%)	2.149	0.542
HE	35 (7.1%)	38 (7.8%)	0.168	0.682
Bacterial peritonitis	237 (48.2%)	229 (47.0%)	0.014	0.565
Portal vein thrombosis	62 (12.8%)	79 (16.7%)	0.037	0.155
**Another clinical character** ***n*** **(%)**			
History of spleen surgery	24 (4.9%)	53 (10.9%)	0.122	**0.000**
MELD Score *n* (%)			6.529	**0.038**
<15	291 (59.4%)	258 (53.1%)		
15–18	72 (14.7%)	100 (20.6%)		
>18	127 (25.9%)	128 (26.3%)		
CTP score M (IQR)	9.00 (8.00, 10.00)	9.00 (8.00, 11.00)	−1.806	0.071
Child-Pugh score *n* (%)			3.233	0.199
A	30 (6.1%)	26 (5.3%)		
B	264 (53.9)	238 (49.0%)		
C	196 (40.0)	222 (45.7%)		

HH, hepatic hydrothorax; *n* (%), the frequency with percentage for categorical variables; M, median; IQR, Interquartile range; UGIB, Upper gastrointestinal bleeding; EGV, Esophageal and gastric fundal varices; HE, Hepatic encephalopathy; History of spleen surgery, splenectomy or splenic embolization. The bold values indicate a statistically significant *p*-value for their analysis of that variable (*p* < 0.05).

### Lab test results in the two groups

The comparison of many indicators between the two groups had statistical significance. Details are as follows: the proportion of patients in the HH group who had low TC (31.0 vs. 22.4%, *P* = 0.002), HDL (58.5 vs. 41.5%, *P* = 0.000), Hb (63.2 vs. 52.7%, *P* = 0.001), PLT (58.3 vs. 34.2%, *P* = 0.018), and PTA (71.0 vs. 60.1%, *P* = 0.000) was higher than in the control group. Compared with individuals without HH, there were more patients with lower IgG (64.9 vs. 72.3%, *P* = 0.012), and higher D-dimer (88.1 vs. 75.4%, *P* = 0.000), CRP (59.5 vs. 48.7%, *P* = 0.001), and PVW (29.2 vs. 21.4%, *P* = 0.005) (Table 2).

**Table 2:** Laboratory test index between the two groups.

**Indicators**	**Normal range**	**Without HH (*N =* 491)**	**HH (*N =* 487)**	**χ2**	***P***
		***N*** **(%)**	***N*** **(%)**		
ALT (U/L)	0–40			0.783	0.376
≤40		264 (53.8%)	276 (56.7%)		
>40		227 (46.2%)	211 (43.3%)		
AST (U/L)	0–40			2.544	0.111
≤40		157 (32.0%)	180 (37.0%)		
>40		334 (68.0%)	307 (63.0%)		
GGT (U/L)	12–64			2.738	0.098
≤64		258 (52.5%)	281 (57.7%)		
>64		233 (47.5%)	307 (42.3%)		
ALB (g/L)	35–55			0.058	0.809
≥35		86 (17.5%)	88 (18.1%)		
<35		405 (82.5%)	399 (81.9%)		
TG (mmol/L)	0.56–1.70			1.535	0.215
≥0.56		415 (84.5%)	399 (81.9%)		
<0.56		76 (15.5%)	88 (18.1%)		
TC (mmol/L)	2.60–6.19			10.015	**0.002**
≥2.60		381 (77.6%)	336 (69.0%)		
<2.60		110 (22.4%)	151 (31.0%)		
HDL (mmol/L)	1.03–1.78			27.811	**<0.001**
≥1.03		287 (58.5%)	202 (41.5%)		
<1.03		204 (41.5%)	285 (58.5%)		
Cr (mmol/L)	50.4–98.1			1.378	0.240
≤98.1		462 (94.1%)	449 (92.2%)		
>98.1		29 (5.9%)	38 (7.8%)		
WBC × 10^9^/L	4–10			1.378	0.240
≤10		462 (94.1%)	449 (92.2%)		
>10		29 (5.9%)	38 (7.8%)		
N × 10^9^/L	0.8–4			2.276	0.131
≤4		382 (77.8%)	359 (73.7%)		
>4		109 (22.2%)	128 (26.3%)		
Hb (g/L)	F:110–150 M:120–160			11.053	**0.001**
≥110 (F)/120 (M)		232 (47.3%)	179 (36.8%)		
<110 (F)/120 (M)		259 (52.7%)	308 (63.2%)		
PLT × 10^9^/L	100–300			5.588	**0.018**
≥100		323 (65.8%)	203 (41.7%)		
<100		168 (34.2%)	284 (58.3%)		
PTA (%)	70–130			13.311	**<0.001**
≥70		196 (39.9%)	141 (29.0%)		
<70		295 (60.1%)	346 (71.0%)		
D-dimer (mg/L)	0–0.5			26.764	**0.000**
≤0.5		121 (24.6%)	58 (11.9%)		
>0.5		370 (75.4%)	429 (88.1%)		
IgG (g/L)	7–16			6.346	**0.012**
≤16		136 (27.7%)	171 (35.1%)		
>16		355 (72.3%)	316 (64.9%)		
IL-6 (pg/mL)	0–7			1.857	0.173
≤7		27 (5.5%)	18 (3.7%)		
>7		464 (94.5%)	469 (96.5%)		
SAA (mg/L)	0–10			2.896	0.089
≤10		197 (40.1%)	170 (34.9%)		
>10		294 (59.9%)	317 (65.1%)		
CRP (ng/L)	0.068–8.2			11.860	**0.001**
≤8.2		252 (51.3%)	197 (40.5%)		
>8.2		239 (48.7%)	290 (59.5%)		
PVW (mm)	6–10			7.927	**0.005**
≤13		386 (78.6%)	345 (70.8%)		
>13		105 (21.4%)	142 (29.2%)		

HH, hepatic hydrothorax; ALT, alanine aminotransferase; AST, aspartate aminotransferase; GGT, γ-glutamyl transpeptidase; ALB, albumin; TG, triglyceride, TC, total cholesterol; HDL, high-density lipoprotein cholesterol, Cr, creatinine, WBC, white blood cell count; N, neutrophil; Hb, hemoglobin; PLT, platelet count, PTA, prothrombin activity; IgG, immunoglobin G; IL-6, interleukin-6; CRP, C reactive protein; SAA, serum amyloid A; PVW, the width of the portal vein. The bold values indicate a statistically significant *p*-value for their analysis of that variable (*p* < 0.05).

### PVW, MELD scores, and CTP scores were the risk factors for HH

**Figure 2:**
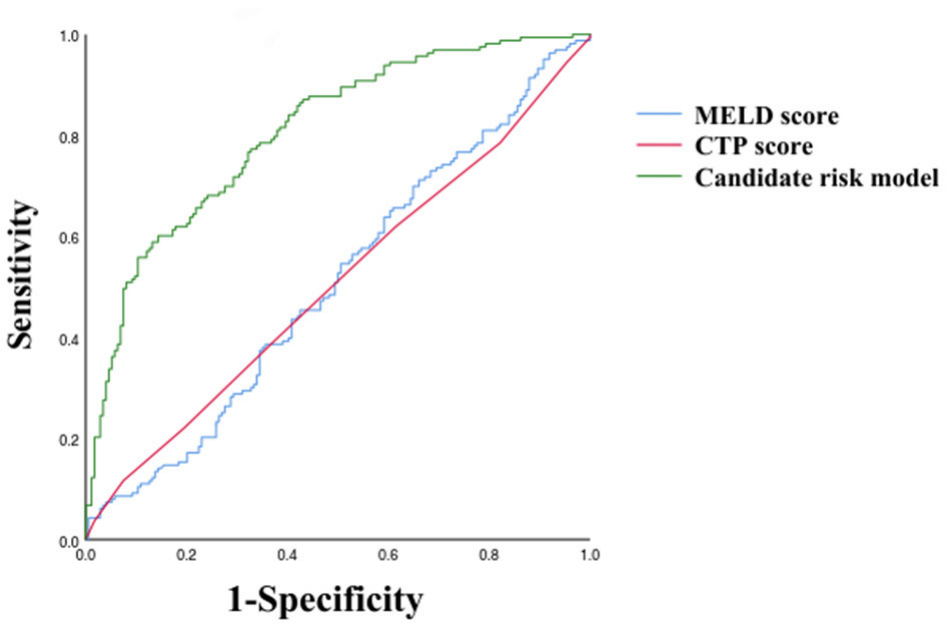
Receiver operating characteristic curves of MELD score, CTP score, and Candidate risk model.

**Figure 3:**
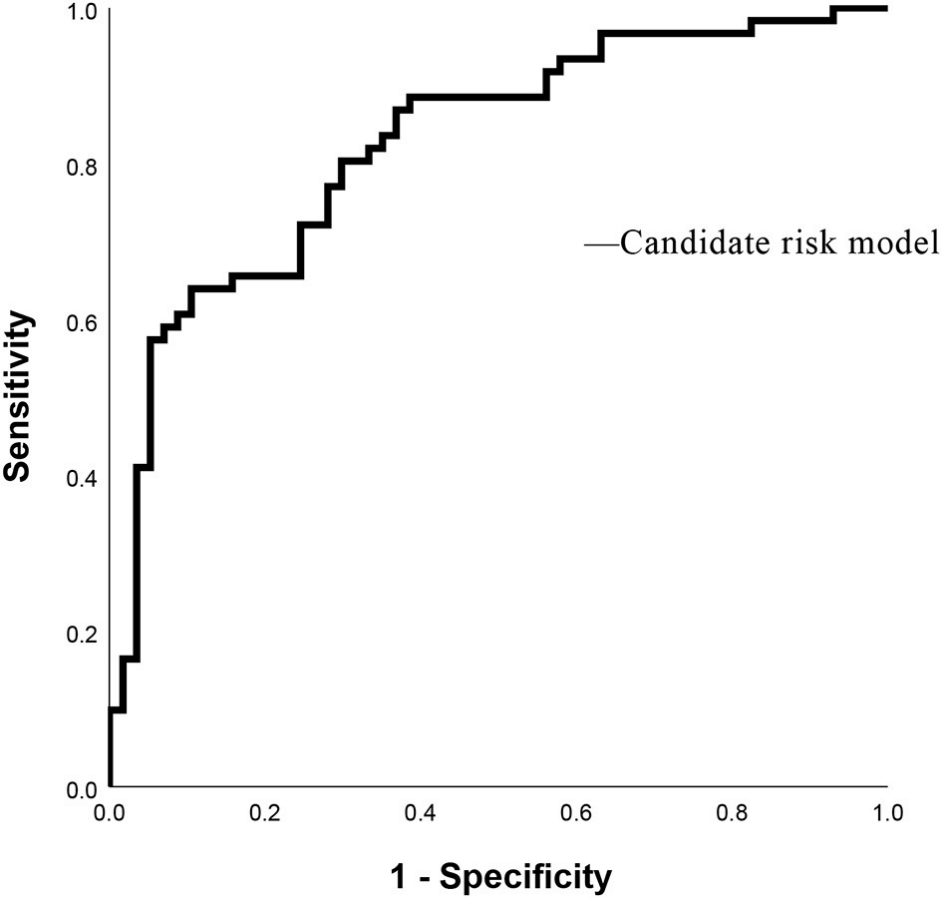
Receiver operating characteristic curves of Candidate risk model in validation group.

As shown in Table 3, PVW (*P* = 0.013, OR = 1.195, 95% CI = 1.038–1.376), PTA (*P* = 0.009, OR = 0.392, 95% CI = 0.194–0.792), D-dimer (*P* = 0.009, OR = 3.822, 95% CI = 1.398–10.450), IgG (*P* = 0.009, OR = 2.127, 95% CI = 1.212–3.735), HDL (*P* = 0.003, OR = 0.384, 95% CI = 0.206–0.716), and MELD scores were significantly associated with the occurrence of HH. Taking the MELD score <15 as the control group, the OR for the occurrence of HH in patients with MELD scores of 15–18 was 4.184, and the OR in the patients with a MELD score of >18 was 4.066. An equation was derived from binary logistic regression in this study as follows: Logit *P* = ln[P/(1-P)] = −24.509 – 0.957^*^(HDL) + 0.755^*^(IgG) – 0.937^*^(PTA) + 1.341^*^(D-dimer) + 0.178^*^(PVW) + 1.431^*^(MELD 15–18) + 1.403^*^(MELD > 18). Comparing the candidate model with the MELD score (AUC = 0.514, 95% CI = 0.452–0.576, *P* = 0.655) and CTP (AUC = 0.511, 95% CI = 0.448–0.573, *P* = 0.736), we found that the AUCs of the candidate risk models were 0.805 (95% CI = 0.758–0.851, *P* < 0.001) (Table 4, Figure 2), and the model's AUCs in the validation group were 0.826 (95% CI = 0.752–0.901, *P* < 0.001) (Table 5, Figure 3).

**Table 3:** Binary logistic regression analysis to identify risk factors for hepatic hydrothorax.

**Indicators**	**Univariate analysis**		**Multivariable analysis**	
	**OR (95%CI)**	***p***	**OR (95%CI)**	***p***
UGIB	0.614 (0.393–0.958)	**0.032**	0.506 (0.191–1.337)	0.170
History of spleen surgery MELD score	0.420 (0.255–0.692)	**0.001**	1.214 (0.418–3.528)	0.721
<15	1.00		1.00	
15–18	1.567 (1.108–2.215)	**0.011**	4.184 (1.465–11.952)	**0.008**
>18	1.137 (0.844–1.530)	0.398	4.066 (1.557–10.621)	**0.004**
TC (mmol/L)	1.590 (0.393–0.653)	**0.002**	0.965 (0.525–1.775)	0.910
HDL (mmol/L)	0.506 (1.532–2.547)	**0.000**	0.384 (0.206–0.716)	**0.003**
Hb (g/L)	1.541 (1.194–1.990)	**0.001**	0.955 (0.447–2.040)	0.906
PLT × 10^9^/L	1.366 (1.054–1.770)	**0.018**	1.918 (0.997–3.692)	0.051
PTA (%)	1.639 (1.256–2.139)	**0.000**	2.552 (1.262–5.161)	**0.009**
D-dimer (mg/L)	2.441 (1.729–3.445)	**0.000**	3.822 (1.398–10.450)	**0.009**
IgG (g/L)	1.417 (1.080–1.858)	**0.012**	2.127 (1.212–3.735)	**0.009**
CRP (ng/L)	1.627 (1.245–2.126)	**0.000**	1.046 (0.542–2.020)	0.893
PVW (mm)	1.079 (1.012–1.151)	**0.02**	1.195 (1.038–1.376)	**0.013**

B, beta, regression coefficient; OR, Odds ratio; 95%CI, Confidence interval; PVW, the width of the portal vein. The bold values indicate a statistically significant *p*-value for their analysis of that variable (*p* < 0.05).

**Table 4:** Receiver operating characteristic analysis for MELD scores, CTP scores, and the candidate risk model in all selected patients.

**Indicators**	**AUC**	**SE**	***P***	**95%CI**	
				**Lower**	**Upper**
MELD Score	0.514	0.032	0.655	0.452	0.576
CTP Score	0.511	0.032	0.736	0.448	0.573
Candidate risk model	0.805	0.024	0.000	0.758	0.851

AUC, Area under curve; 95%CI, Confidence interval; SE, Standard error.

**Table 5:** Receiver operating characteristic analysis for the candidate risk model in the internal validation group.

**Indicators**	**AUC**	**SE**	***P***	**95%CI**	
				**Lower**	**Upper**
Candidate risk model	0.826	0.038	0.000	0.752	0.901

AUC, Area under curve; 95%CI, Confidence interval; SE, Standard error.

### The incidence of portal vein thrombosis was higher in bilateral pleural effusion compared with left and right pleural effusions

Among 487 cases with HH, there were 99 cases with left pleural effusions (20.4%), 217 cases with right (44.5%) pleural effusions, and 171 cases with bilateral pleural effusions (35.1%). Clinical characteristics and lab test results shown in Tables 6, 7 were analyzed to explore differences between the three groups. The results showed that the incidence of portal vein thrombosis was higher in bilateral pleural effusion compared with left and right pleural effusions (*P* = 0.018).

**Table 6:** Clinical characteristics among left, right, and bilateral pleural effusions.

**Variables**	**Left**	**Right**	**Bilateral**	**H/χ2**	***P***
	***N** =* **99**	***N** =* **217**	***N** =* **171**		
Sex (male/female) n	72/27	154/63	130/41	0.055	0.973
Age *n* (%)				1.963	0.742
<40	11 (11.1%)	17 (7.8%)	18 (10.5%)		
40–60	57 (57.6%)	121 (55.8%)	89 (52%)		
>60	31 (31.3%)	79 (36.4%)	64 (37.4%)		
Etiology n (%)				15.015	0.241
HBV	70 (73.7%)	170 (78.3%)	109 (63.7%)		
HCV	1 (1.1%)	4 (1.8%)	3 (1.8%)		
PBC	3 (3.2%)	8 (3.7%)	9 (5.3%)		
AIH	1 (1.1%)	1 (0.5%)	4 (2.3%)		
Alcoholic Liver	8 (8.4%)	20 (9.2%)	23 (13.5%)		
Unknown Reason	16 (16.2%)	14 (6.4%)	17 (10.0%)		
**Complication/combination disease n (%)**					
Hypertension	29 (29.6%)	47 (21.7%)	32 (18.7%)	4.337	0.114
Diabetes	19 (19.4%)	28 (12.9%)	32 (18.7%)	3.257	0.196
UGIB	12 (12.2%)	16 (7.4%)	25 (14.6%)	5.396	0.067
EGV	35 (35.7%)	73 (33.6%)	57 (33.3%)	0.286	0.867
HE	6 (6.1%)	16 (7.4%)	16 (9.4%)	4.939	0.552
Bacterial peritonitis	51 (52.0%)	95 (43.8%)	82 (48%)	1.965	0.374
Portal vein thrombosis	9 (9.4%)	32 (15.1%)	37 (22.4%)	8.048	**0.018**
Liver cancer	17 (17.3%)	48 (22.2%)	20.80%	5.826	0.667
**Another clinical character n (%)**					
History of spleen surgery	16 (16.3%)	19 (8.8%)	18 (10.5%)	4.022	0.134
MELD Score *n* (%)				2.868	0.580
<15	72 (73.5%)	164 (75.6%)	120 (70.1%)		
15–18	14 (14.3%)	27 (12.4%)	20 (11.7%)		
>18	12 (12.2%)	26 (12.0%)	31 (18.2%)		
CTP score M (IQR)	9.00 (8.00, 11.00)	9.00 (8.00, 11.00)	9.50 (8.00, 11.00)	2.036	0.361
Child-Pugh score				8.658	0.070
A	6 (6.1%)	13 (6.0%)	7 (4.1%)		
B	50 (50.5%)	113 (52.3%)	68 (39.8%)		
C	43 (43.4%)	90 (41.7%)	96 (56.1%)		

HH, hepatic hydrothorax; *n* (%), the frequency with percentage for categorical variables; M, median; IQR, Interquartile range; UGIB, Upper gastrointestinal bleeding; EGV, Esophageal and gastric fundal varices; HE, Hepatic encephalopathy; History of spleen surgery, splenectomy or splenic embolization. The bold values indicate a statistically significant *p*-value for their analysis of that variable (*p* < 0.05).

**Table 7:** Laboratory test index among left, right, and bilateral pleural effusions.

**Variables**	**Normal range**	**Left (*N =* 99)**	**Right (*N =* 217)**	**Bilateral (*N =* 171)**	**H**	***P***
		**Median (IQR)**	**Median (IQR)**	**Median (IQR)**		
ALT (U/L)	0–40	34.00 (21.00, 59.00)	34.50 (20.00, 64.50)	34.50 (19.75, 82.00)	0.055	0.973
AST (U/L)	0–40	49.00 (30.00, 83.00)	54.00 (33.00, 91.00)	51.00 (29.00, 122.00)	0.977	0.614
GGT (U/L)	12–64	47.00 (24.00, 104.00)	51.00 (23.00, 108.50)	57.00 (27.00, 130.00)	1.350	0.509
ALB (g/L)	35–55	28.20 (24.60, 33.60)	28.30 (24.95, 32.90)	28.50 (24.60, 32.40)	0.037	0.982
TG (mmol/L)	0.56–1.70	0, 875 (0.658, 1.175)	0.780 (0.610, 1.015)	0.825 (0.620, 1.150)	5.886	0.053
TC (mmol/L)	2.60–6.19	3.01 (2.373, 3.84)	3.27 (2.34, 3.93)	3.06 (2.45, 3.90)	1.234	0.539
HDL (mmol/L)	1.03–1.78	0.89 (0.65, 1.18)	0.97 (0.64, 1.32)	0.90 (0.55, 1.22)	3.766	0.152
Cr (umol/L)	50.4–98.1	63.60 (54.30, 74.00)	62.10 (52.80, 75.00)	63.20 (54.50, 73.40)	1.143	0.565
WBC × 10^9^/L	4–10	4.12 (2.86, 6.54)	3.97 (2.89, 5.76)	4.38 (3.09, 6.11)	2.083	0.353
N × 10^9^/L	0.8–4	2.70 (1.60, 4.43)	2.43 (1.60, 3.80)	2.76 (1.88, 4.60)	2.791	0.248
Hb (g/L)	110–150	109.00 (87.00, 125.00)	110.00 (90.50, 125.00)	109.00 (85.00, 126.00)	0.257	0.879
PLT × 10?/L	100–300	86.00 (50.00, 147.00)	87.00 (53.50, 132.00)	87.00 (56.00, 137.00)	0.328	0.849
PTA (%)	70–130	60.00 (49.00, 73.00)	61.00 (49.00, 70.50)	62.00 (50.75, 73.00)	0.520	0.771
IgG (g/L)	7–16	14.07 (10.62, 18.87)	16.63 (13.08, 20.29)	15.33 (11.54, 19.83)	5.967	0.051
IL-6 (pg/mL)	0–7	27.78 (15.59, 64.80)	30.79 (13.95, 71.34)	33.90 (5.46, 1, 519)	0.647	0.724
SAA (mg/L)	0–10	13.60 (5.13, 77.35)	14.00 (4.23, 53.65)	12.90 (4.33, 55.15)	0.450	0.798
CRP (ng/L)	0.068–8.2	10.89 (3.82, 36.26)	10.29 (3.35, 24.96)	12.36 (4.88, 35.17)	3.821	0.148
PVW (mm)	6–10	12.00 (12.00, 15.00)	12.00 (12.00, 14.00)	13.00 (12.00, 14.00)	1.041	0.594

PVW, the width of the portal vein; IQR, Interquartile range.

However, it is important to note that the abovementioned results are not meant to be used for the minority of patients who develop HH without developing ascites.

## Discussion

The patients who developed severe complications of cirrhosis usually presented clinical manifestations of chest tightness, shortness of breath, cough, nausea, and, in a few patients, severe dyspnea and tachycardia. There was no significant difference in presentation from the diseases in the exclusion criteria, thus, the abovementioned exclusion criteria needed to be added to the diagnosis to exclude the relevant patients who did not meet the requirements of this study. Other than that, HH has a close relationship with a poor prognosis (23, 24). Therefore, our research aimed to explore the risk factors of hepatic hydrothorax for cirrhotic patients, which can help clinicians to develop secondary prevention strategies and improve the patient's prognosis. The MELD score is an exact predictive model suitable for patients with liver cirrhosis at the end stage and is extensively applied to assess liver disease severity and mortality (17, 18). The result of the univariate analysis showed that patients had a significant difference in MELD scores between the two groups. Combined with the findings of Hou et al., it is possible that the incidence of HH is strongly associated with the severity degree of cirrhosis (21). Similarly, the result of the logistics regression also found that a higher MELD score is a risk factor for HH.

The association of the MELD score and HH supports other findings of the binary logistic regression. For patients with liver cirrhosis, lower HDL and PTA and higher PVW, D-dimer, and IgG also correlate with the development of HH. The explanations are as follows: (1) HDL, IgG, and PTA: These three can indicate the degree of liver function impairment. First, HDL production decreased because of the liver's decreased synthesis of apolipoproteins A and B (25, 26). Some studies suggested that HDL-cholesterol is a reliable predictor of the liver's function and of disease progression in chronic liver failure (27–29). IgG is an antibody produced by plasma cells that are transformed from B lymphocytes when stimulated by antigens from bacteria, viruses, and toxins and can specifically bind to antigens (30). In patients with chronic hepatitis, humoral immunity is activated, resulting in elevated IgG in the body. Therefore, a higher IgG also means more severe hepatitis and cirrhosis (31, 32). PTA calculated by PT reflects the activity of blood coagulation factorsI, II, V, VII, and X. Both the prolongation of PT and the decrease of PTA are due to liver impairment and reduced synthesis of coagulation factors. Hence, PTA is also thought to be a key predictor of liver function (33, 34).

Therefore, we conclude that because the three factors mentioned above can indicate the severity of cirrhosis and the impairment of the liver's synthetic function, they have a strong association with the occurrence of HH, which is in line with the findings of Mouelhi et al. (35). (2) D-dimer: The D-dimer of the patients with HH was significantly higher than those in the control group. Since D-dimer has a positive correlation with both portal hypertension and the MELD score (36–39), the odds of HH increase with a higher D-dimer. (3) PVW: An increased width of the portal vein means elevated portal vein pressure. Some studies suggested that it may result in the high pressure of vena azygos, and then the lymph and other body fluids will leak into the pleural cavity (40, 41).

However, in terms of risk factors for HH, our study differed slightly from the conclusions of previous studies. Hou et al. stated that HH was positively associated with moderate-large ascites, Child–Pugh class B–C, lower albumin, higher prothrombin time, and the international normalized ratio (21). Deleuran et al. and Matei et al. indicated that bilirubin, diabetes, and the non-use of non-selective beta-blockers were risk factors for hepatic hydrothorax (7, 22). These differences probably relate to the following points: (1) the patients in our study were all from Asia, and the etiology of cirrhosis was mostly HBV, which was different from the context of other studies and (2) different types of studies may cause diverse results.

By comparing lab data among left, right, and bilateral pleural effusions, the right side is a more common distribution, which is similar to the conclusion of the previous studies (4, 42, 43). In addition to the hypothesis of the physiological defect in the diaphragm, Huang et al. suggested that it is likely due to the close anatomical relationship of bare areas of the liver with the diaphragm and the fact that the left side of the diaphragm is thicker and more muscular than the right side (44). In addition to the aforementioned finding, we have another conclusion: The severity of liver cirrhosis has no relationship with the distribution of pleural effusion, and portal vein thrombosis is more common in cirrhotic patients with bilateral pleural effusion compared to those with unilateral pleural effusion, but the mechanism is still unknown. The fact that portal vein thrombosis further aggravates portal hypertension, resulting in increased parietal pleural pressure in the umbilical and hemiazygos veins, may be the main factor.

Limitations of the study are as follows: (1) In choosing samples, retrospective studies are more prone to selection bias, but the inclusion of a larger sample size in our study mitigated this effect. (2) We have only studied the risk factors for the development of HH in cirrhosis patients with ascites. Whether our conclusion applies to the entire cirrhotic population, the answer is unknown. (3) In fact, we collected data on the depth of ascites as a criterion to assess the severity of ascites that were reported in the patient's abdominal imaging findings. However, the univariate analysis of this variable was not statistically significant, which may be related to the fact that this value only reflected the depth of ascites in some anatomical planes of the patient's abdomen, and it could not accurately reflect the severity of the patient's ascites. Therefore, we did not take it as a variable.

## Conclusion

It was found that lower HDL and PTA and higher PVW, D-dimer, IgG, and MELD scores have a strong correlation with the development of hepatic hydrothorax. Moreover, portal vein thrombosis is more common in cirrhotic patients with bilateral pleural effusion compared to those with unilateral pleural effusion.

## Data availability statement

The original contributions presented in the study are included in the article/supplementary material, further inquiries can be directed to the corresponding author.

## Ethics statement

This study was approved by the Ethics Committee of Shandong Public Health Clinical Center (SPHCC-2021-16). The patients/participants provided their written informed consent to participate in this study. Written informed consent was obtained from the individual(s) for the publication of any potentially identifiable images or data included in this article.

## Author contributions

WD: study concept and design and critical revision of the manuscript for important intellectual content. XB and XL: acquisition of data. XB and XL: analysis and interpretation of data and statistical analysis. XB: drafting of the manuscript. YS and WL: administrative, technical, or material support. QL: study supervision. All authors read and approved the final manuscript.
